# Supplementary material for: Blocking sense‐strand activity improves potency, safety and specificity of anti‐hepatitis B virus short hairpin RNA
Source: EMBO Mol Med. 2016 Jul 29;8(9):1082–98. doi: 10.15252/emmm.201506172 (PMC5009812; doi:10.15252/emmm.201506172)
Supplement: Supplementary file 1 — Appendix [file EMMM-8-1082-s001.pdf]

Appendix for:

Blocking sense strand activity improves potency, safety and specificity of anti-hepatitis B virus short hairpin RNA

Thomas Michler, Stefanie Große, Stefan Mockenhaupt, Natalie Röder, Ferdinand Stückler, Bettina Knapp, Chunkyu Ko, Mathias Heikenwalder, Ulrike Protzer, Dirk Grimm

Contents:

Appendix Supplementary Discussion

Appendix Supplementary Methods

Appendix Supplementary References

Appendix Figures S1-S2

Appendix Tables S1-S5

Appendix Supplementary Discussion

As noted in the main text, we are careful when interpreting complex data on *in vivo* liver gene expression and when assigning dysregulated genes as direct shRNA off-targets, for four reasons. First, roughly half of our genes (even two thirds in (Maczuga *et al*, 2014)) were up- rather than down-regulated, yet only the latter is expected for a direct shRNA (off-)target. Second, although we found seed matches for the shRNA sense strand in over 80% of full transcripts of significantly down-regulated genes, the number was lower when we exclusively analysed the 3'UTR (12.2%; still higher than background or up-regulated genes, Appendix Table S2). Important to note here is that prior to our study, data on *in vivo* specificity of shRNAs were extremely sparse. Therefore, the rules that determine on- versus off-targeting in whole organisms are far from clear, as is the question which algorithms hold the highest predictive value (Birmingham *et al*, 2006; Maczuga *et al*, 2013). Our new data and vectors should help to shed light on the underlying mechanisms and thus foster the implementation of more specific next-generation RNAi therapeutics. Third, as noted, the degree of down-regulation amongst all dysregulated genes with shHBV7 sense-strand seed match (irrespective of significance) was small. This was actually expected since we had aimed at pre-minimising toxicity, by using moderate AAV doses and the weak H1 promoter for shRNA expression in all our constructs. The overall mild degree of toxicity with no significant pathohistological changes seen in livers harvested 84 days post-AAV treatment (Fig 5E) was also confirmed in livers from day 15 post-AAV treatment using Hematoxylin/Eosin, ki67 (proliferation marker) or Caspase-3 (apoptosis marker) staining, none of which showed notable abnormalities (Fig EV4). Further noteworthy in

this context is that shRNA off-targeting likely predominantly occurs via imperfect target binding beyond the seed region, which triggers miRNA-like inhibition on mRNA levels in a range of two-fold or less (congruent with our observations). As these subtle changes might be missed in DNA microarray analyses, future studies should include protein arrays to more comprehensively detect all direct and indirect off-targets.

Fourth and last, it is well possible that most dysregulated genes (especially those that were up-regulated) that we detected 15 days after vector administration were actually secondary or later hits, resulting from earlier perturbance of other genes and compensatory reactions of cellular networks. In addition, a meta-analysis of published RNAi experiments (Khan *et al*, 2009) suggests that shRNA-mediated RISC saturation and miRNA dysregulation can largely and widely alter gene expression profiles, including both up- and down-regulation as noted here. One line of evidence for this more general mechanism could be our pathway analysis in Appendix Table S4 which shows that many of the dysregulated genes are involved in steroid metabolism. The latter is regulated by miRNAs in the liver (Rottiers & Naar, 2012) and is altered upon interference with the predominant liver miRNA miR-122 (Esau *et al*, 2006). We have previously shown that both the expression and function of miR-122 are impaired in cells and mice that express high levels of shRNAs (Grimm *et al*, 2006), which could support this model. Further consistent is that genes involved in steroid metabolism were not dysregulated by the shRNA/TuD, shRNA/Ago2 or the miHBV7 vector, which may support a beneficial effect of all three strategies on RISC/miRNA activity. Finally, many other dysregulated genes that we detected in our mice control essential functions such as cell death/survival, cell morphology and cell cycle. Such hits were also observed by

Maczuga and co-workers (Maczuga *et al*, 2014) and may generally reflect cellular toxicity rather than representing direct shRNA off-targets.

Appendix Supplementary Methods

Pathohistological analysis

Sections (2 µm) of livers (fixed in 4% paraformaldehyde and paraffin-embedded) were stained with Hematoxylin/Eosin or anti-ki67 (NeoMarkers/Thermo Scientific, Waltham, Massachusetts, USA; RM-9106-S1; rabbit anti-mouse antibodies; retrieval at 95°C with EDTA for 30 min; 1:200 solution) or anti-Cleaved Caspase 3 (Cell Signaling, Leiden, The Netherlands; #9661; rabbit anti-mouse; retrieval at 100°C for 20 min with EDTA; 1:300 solution) antibodies (Wolf *et al*, 2014). Incubation in Ventana buffer and staining was performed on a NEXES immunohistochemistry robot (Ventana Instruments, Tucson, Arizona, USA) using an IVIEW DAB Detection Kit (Ventana) or on a Bond MAX (Leica, Wetzlar, Germany). For analysis, slides were scanned using a SCN 400 slide scanner (Leica).

PCR

For analysis of HBV RNA from liver lysate, RNA was extracted from 30 mg liver tissue with the RNeasy mini kit (Qiagen) and cDNA synthesized with the Superscript III kit (Thermo Fisher Scientific). HBV transcripts were amplified with primers specific for only the 3.5 kb transcripts, or with primers binding to the common 3´ end of all HBV transcripts (Yan *et al*, 2012). Results were normalised to two housekeeping genes (Hypoxanthine-guanine phosphoribosyltransferase (HPRT) and cyclophylin). To analyse

viremia in HBV-transgenic mice, DNA was extracted from 20 µl serum with the High Pure Viral Nucleic Acid Kit (Roche Diagnostics) and amplified with primers HBV 1844 and HBV 1745. All PCRs were performed on a LightCycler 480 (Roche Diagnostics) using the primers and PCR conditions shown in Appendix Table S5.

Appendix Supplementary References

Birmingham A, Anderson EM, Reynolds A, Ilsley-Tyree D, Leake D, Fedorov Y, Baskerville S, Maksimova E, Robinson K, Karpilow J, Marshall WS, Khvorova A (2006) 3' UTR seed matches, but not overall identity, are associated with RNAi off-targets. *Nat Methods* **3:** 199-204

Esau C, Davis S, Murray SF, Yu XX, Pandey SK, Pear M, Watts L, Booten SL, Graham M, McKay R, Subramaniam A, Propp S, Lollo BA, Freier S, Bennett CF, Bhanot S, Monia BP (2006) miR-122 regulation of lipid metabolism revealed by in vivo antisense targeting. *Cell Metab* **3:** 87-98

Grimm D, Streetz KL, Jopling CL, Storm TA, Pandey K, Davis CR, Marion P, Salazar F, Kay MA (2006) Fatality in mice due to oversaturation of cellular microRNA/short hairpin RNA pathways. *Nature* **441:** 537–541

Khan AA, Betel D, Miller ML, Sander C, Leslie CS, Marks DS (2009) Transfection of small RNAs globally perturbs gene regulation by endogenous microRNAs. *Nat Biotechnol* **27:** 549-555

Maczuga P, Lubelski J, van Logtenstein R, Borel F, Blits B, Fakkert E, Costessi A, Butler D, van Deventer S, Petry H, Koornneef A, Konstantinova P (2013) Embedding siRNA sequences targeting apolipoprotein B100 in shRNA and miRNA scaffolds results in differential processing and in vivo efficacy. *Mol Ther* **21:** 217-227

Maczuga P, Verheij J, van der Loos C, van Logtenstein R, Hooijer G, Martier R, Borel F, Lubelski J, Koornneef A, Blits B, van Deventer S, Petry H, Konstantinova P (2014) Therapeutic expression of hairpins targeting apolipoprotein B100 induces phenotypic and transcriptome changes in murine liver. *Gene Ther* **21:** 60-70

Rottiers V, Naar AM (2012) MicroRNAs in metabolism and metabolic disorders. *Nat Rev Mol Cell Biol* **13:** 239-250

Wolf MJ, Adili A, Piotrowitz K, Abdullah Z, Boege Y, Stemmer K, Ringelhan M, Simonavicius N, Egger M, Wohlleber D, Lorentzen A, Einer C, Schulz S, Clavel T, Protzer U, Thiele C, Zischka H, Moch H, Tschop M, Tumanov AV et al (2014) Metabolic

activation of intrahepatic CD8+ T cells and NKT cells causes nonalcoholic steatohepatitis and liver cancer via cross-talk with hepatocytes. *Cancer Cell* **26:** 549-564

Yan H, Zhong G, Xu G, He W, Jing Z, Gao Z, Huang Y, Qi Y, Peng B, Wang H, Fu L, Song M, Chen P, Gao W, Ren B, Sun Y, Cai T, Feng X, Sui J, Li W (2012) Sodium taurocholate cotransporting polypeptide is a functional receptor for human hepatitis B and D virus. *Elife* **1:** e00049

Appendix Figures and Tables

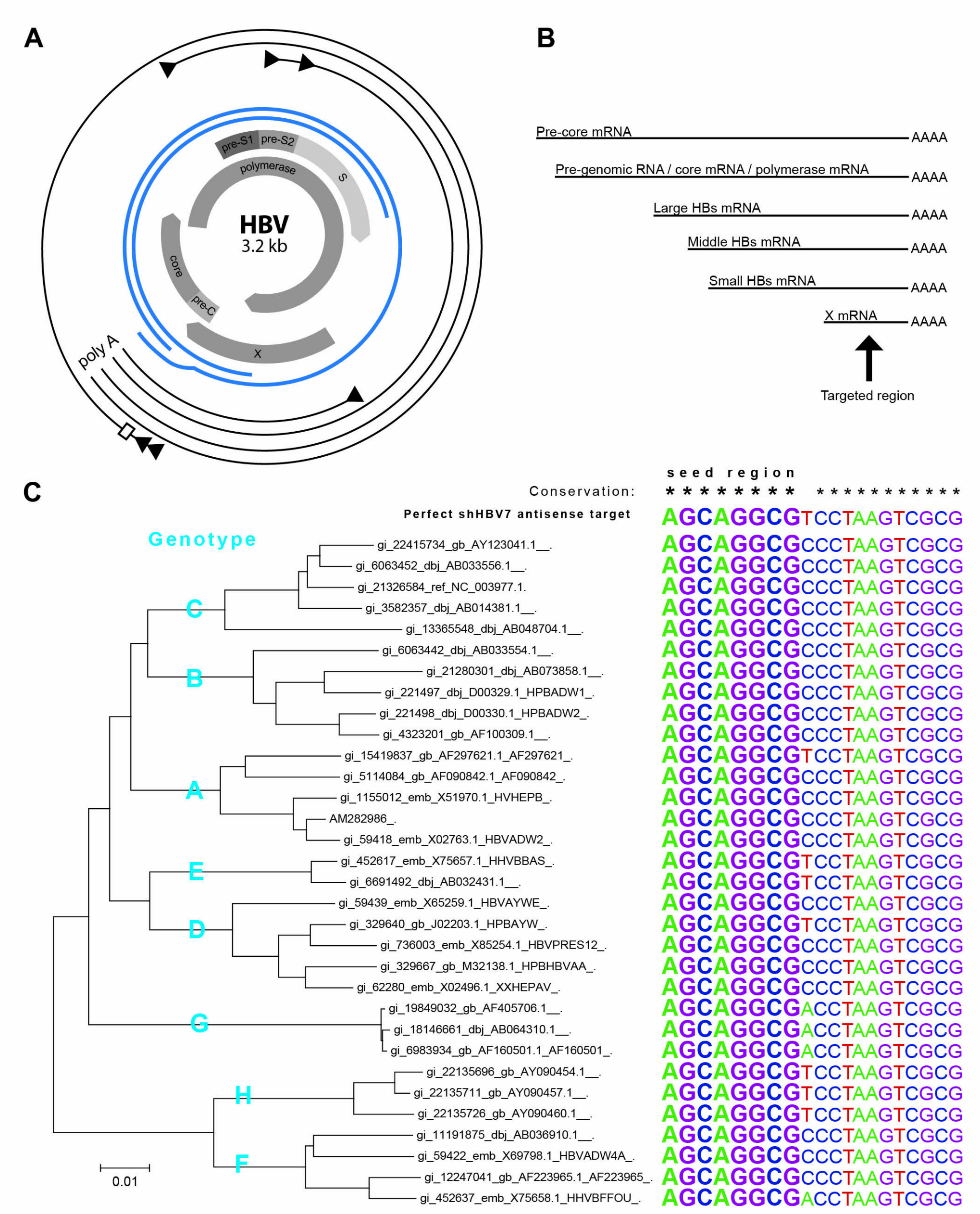

Appendix Figure S1 - Location and conservation of selected shHBV7 binding site.

- A Schematic HBV genome. Grey arrows in the center indicate open reading frames. Blue lines denote the partially double-stranded DNA genome. Thin outer lines symbolise transcripts, with arrows showing transcriptional start sites. The white box represents the RNA encapsidation signal.
- B Alignment of all HBV transcripts and indication of shHBV7 target region.
- C Alignment of shHBV7 with all HBV genotypes. The shRNA seed region, *i.e.*, nt 1 to 8 from the 5' end, is highlighted in capital letters. Note how this seed region, whose binding to the target site is most important for shRNA specificity and activity, is 100% conserved across all HBV genotypes. The bar on the bottom left shows the p-distance.

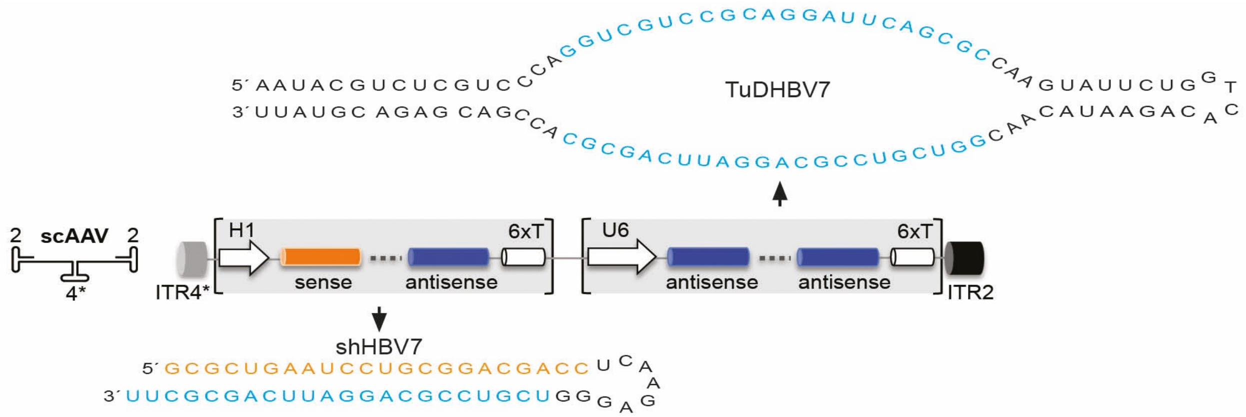

Appendix Figure S2 - **Structure of the dual AAV vector (center).**
Shown on top is the U6 promoter-driven TuD with two perfect binding sites (blue) for the shHBV7 sense strand. Depicted at the bottom is the H1 promoter-driven anti-HBV shHBV7 (sense strand in orange, antisense strand in blue).

Figure	Panel(s)	Treatment group(s)	n	p value	*
Fig. 2	A - C	all groups	3		
	A left	empty vector vs. shHBV4		< 0.0001	****
		empty vector vs. shHBV5		< 0.0001	****
		empty vector vs. shHBV6		< 0.0001	****
		empty vector vs. shHBV7		< 0.0001	****
	A right	empty vector vs. shHBV4		< 0.0001	****
		empty vector vs. shHBV5		< 0.0001	****
		empty vector vs. shHBV6		0.0002	***
		empty vector vs. shHBV7		< 0.0001	****
	B	empty vector vs. shHBV4		0.0001	***
		empty vector vs. shHBV6		0.0007	***
		empty vector vs. shHBV7		< 0.0001	****
	C left	empty vector vs. shHBV4		0.0022	**
		empty vector vs. shHBV7		0.0006	***
	C right	empty vector vs. shHBV4		0.0079	**
		empty vector vs. shHBV5		< 0.0001	****
		empty vector vs. shHBV7		0.0037	**
	D	all groups	1		
Fig. 3	B	all groups	3		
		shHBV7/TuDα1AT sense bs vs. antisense bs		0.0001	***
		shHBV7/TuDα1AT sense bs vs. no bs		< 0.0001	****
		shHBV7/TuDα1AT antisense bs vs. no bs		< 0.0001	****
		shHBV7/TuDα1AT sense bs vs. shHBV7/TuDHBV7 sense bs		0.0001	***
		shHBV7/TuDHBV7 sense bs vs. antisense bs		< 0.0001	****
		shHBV7/TuDHBV7 antisense bs vs. no bs		< 0.0001	****
	C left and right	shα1AT	5		
		shα1AT/TuDα1AT	5		
		shα1AT/TuDHBV7	9		
		shHBV7	14		
		shHBV7/TuDα1AT	7		
		shHBV7/TuDHBV7	14		
	C left	d5 shHBV7/TuDHBV7 vs. shHBV7		0.0004	***
		d5 shHBV7/TuDHBV7 vs. shHBV7/TuDα1AT		0.0013	**
		d10 shHBV7/TuDHBV7 vs. shHBV7		< 0.0001	****
		d10 shHBV7/TuDHBV7 vs. shHBV7/TuDα1AT		0.0017	**
		d15 shHBV7/TuDHBV7 vs. shHBV7		0.0005	***
		d15 shHBV7/TuDHBV7 vs. shHBV7/TuDα1AT		0.0242	*
	C right	d5 shHBV7/TuDHBV7 vs. shHBV7		0.0018	**
		d5 shHBV7/TuDHBV7 vs. shHBV7/TuDα1AT		< 0.0001	****
		d10 shHBV7/TuDHBV7 vs. shHBV7		0.0307	*
		d10 shHBV7/TuDHBV7 vs. shHBV7/TuDα1AT		0.0319	*
Fig. 4	A - C	all groups	3		
	A left	empty vector vs. shHBV7		< 0.0001	****
		empty vector vs. shHBV7/TuDHBV7		< 0.0001	****
		empty vector vs. shHBV7/Ago2		< 0.0001	****
		empty vector vs. miHBV7		< 0.0001	****
	A right	empty vector vs. shHBV7		< 0.0001	****
		empty vector vs. shHBV7/TuDHBV7		< 0.0001	****
		empty vector vs. shHBV7/Ago2		< 0.0001	****
		empty vector vs. miHBV7		< 0.0001	****
	B	empty vector vs. shHBV7		< 0.0001	****
		empty vector vs. shHBV7/TuDHBV7		< 0.0001	****
		empty vector vs. shHBV7/Ago2		< 0.0001	****
		empty vector vs. miHBV7		< 0.0001	****
	C left	empty vector vs. shHBV7		< 0.0001	****
		empty vector vs. shHBV7/TuDHBV7		< 0.0001	****
		empty vector vs. shHBV7/Ago2		0.0001	***
		empty vector vs. miHBV7		0.0003	***
	C right	empty vector vs. shHBV7		0.0005	***
		empty vector vs. shHBV7/TuDHBV7		0.0001	***
		empty vector vs. shHBV7/Ago2		0.0022	**
		empty vector vs. miHBV7		0.0017	**
	D	all groups	1		

Fig. 5	B-D	shα1AT	5		
		shHBV7	6		
		shHBV7/TuDHBV7	6		
		shHBV7/Ago2	5		
		miHBV7	5		
	B	d7 shHBV7 vs. shα1AT		0.0377	*
		d7 shHBV7 vs. miHBV7		0.0001	***
		d7 shHBV7 vs. shHBV7/TuDHBV7		< 0.0001	****
		d14 shHBV7 vs. shα1AT		0.0050	**
		d14 shHBV7 vs. miHBV7		0.0311	*
		d14 shHBV7 vs. shHBV7/TuDHBV7		0.0002	***
		d21 shHBV7 vs. shα1AT		0.0040	**
		d21 shHBV7 vs. miHBV7		0.0132	*
		d21 shHBV7 vs. shHBV7/TuDHBV7		0.0465	*
		d28 shHBV7 vs. shα1AT		0.0025	**
		d28 shHBV7 vs. miHBV7		0.0278	*
		d56 shHBV7 vs. shα1AT		0.0445	*
		d84 shHBV7 vs. shHBV7/TuDHBV7		0.0161	*
	C	shHBV7 vs. miHBV7		< 0.0001	****
		shHBV7 vs. shHBV7/TuDHBV7		< 0.0001	****
		shHBV7 vs. shHBV7/Ago2		< 0.0001	****
	D	shHBV7 vs. miHBV7		0.0038	**
		shHBV7 vs. shHBV7/TuDHBV7		0.0116	*
Fig. 6	A	Mock	5		
		empty vector	6		
		shHBV7	6		
		shHBV7/TuDHBV7	6		
		shHBV7/Ago2	6		
		miHBV7	6		
		d5 shHBV7/TuDHBV7 vs. shHBV7		0.0249	*
		d5 shHBV7/TuDHBV7 vs. shHBV7/Ago2		0.0004	***
		d5 shHBV7/TuDHBV7 vs. miHBV7		0.0028	**
		d10 shHBV7/TuDHBV7 vs. shHBV7		< 0.0001	****
		d10 shHBV7/TuDHBV7 vs. shHBV7/Ago2		0.0437	*
		d10 shHBV7/TuDHBV7 vs. miHBV7		0.0017	**
		d15 shHBV7/TuDHBV7 vs. shHBV7		0.0017	**
		d15 shHBV7/TuDHBV7 vs. miHBV7		0.0033	**
	C	sense seed match: down-regulated vs. all genes on microArray		0.0408	*
	D	no shHBV7 seed match	15065		
		shHBV7 sense seed match	1658		
		shHBV7 antisense seed match	328		
		sense seed match: shHBV7/TuDHBV7 vs. shHBV7		0.0187	*
		sense seed match: shHBV7/TuDHBV7 v.s. shHBV7/Ago2		0.0038	**
		sense seed match: shHBV7/TuDHBV7 v.s. miHBV7		0.0305	*

**EV2**	**A**	shα1AT	5		
		shHBV7	5		
		shHBV7/TuDHBV7	5		
		shHBV7/Ago2	4		
		miHBV7	5		
		shα1AT vs. shHBV7		0.0082	**
		shα1AT vs. shHBV7/TuDHBV7		0.0021	**
		shα1AT vs. shHBV7/Ago2		0.0024	**
		shα1AT vs. miHBV7		0.0356	*
	**B left and right**	shα1AT	4		
		shHBV7	6		
		shHBV7/TuDHBV7	6		
		shHBV7/Ago2	6		
		miHBV7	6		
	**B left**	shα1AT vs. shHBV7		< 0.0001	****
		shα1AT vs. shHBV7/TuDHBV7		< 0.0001	****
		shα1AT vs. shHBV7/Ago2		< 0.0001	****
		shα1AT vs. miHBV7		< 0.0001	****
	**B right**	shα1AT vs. shHBV7		< 0.0001	****
		shα1AT vs. shHBV7/TuDHBV7		< 0.0001	****
		shα1AT vs. shHBV7/Ago2		< 0.0001	****
		shα1AT vs. miHBV7		< 0.0001	****
		shHBV7 vs. miHBV7		0.0012	**
		shHBV7/TuDHBV7 vs. miHBV7		0.0020	**
**EV3**		Mock	5		
		Empty vector	4		
		shHBV7	4		
		shHBV7/TuDHBV7	6		
		shHBV7/Ago2	5		
		miHBV7	6		
		Mock vs. Empty vector		0.0389	*
		Mock vs. shHBV7		< 0.0001	****
		Empty vector vs. shHBV7		0.0065	**
		shHBV7 vs. shHBV7/TuDHBV7		0.0340	*
		shHBV7 vs. shHBV7/Ago2		0.0217	*
		shHBV7 vs. miHBV7		0.0027	**

Appendix Table S1 - Number of biological replicates (n) and exact p values.
vs., versus.

		All genes on chip	Genes dysregulated by shHBV7	
			Downregulated	Upregulated
	Number of genes	26,515	67	59
Whole transcript	Genes with analysis	21,425	51	47
	Sense (%)	14,100 (65.8%)	41 (80.4%)	31 (66.0%)
	Antisense (%)	6,690 (31.2%)	22 (43.1%)	15 (32.0%)
3´UTR	Genes with analysis	18,919	49	43
	Sense (%)	1,933 (10.2%)	6 (12.2%)	3 (7.0%)
	Antisense (%)	388 (2.0%)	1 (2.0%)	1 (2.3%)

Appendix Table S2 - **Frequency of shHBV7 2-7 nt seed matches in whole transcripts or 3'UTRs of dysregulated genes, and background frequency.**
Shown are data for all genes represented on the Affymetrix Mouse Gene 2.0 ST Array. Dysregulated genes in shHBV7-treated animals (compared to mock; adjusted p<0.25) were identified as explained in Methods. Absolute numbers of genes with at least one 2-7 nt seed match for the shHBV7 sense or antisense strand are shown with frequencies across all genes that could be analysed in brackets.

UP-REGULATED

ID (Affymetrix)	SYMBOL	log2 fold change	p-value	adusted p-value	whole transcript		3´ UTR	
					sense seed match (2-7nt)	antisense seed match (2-7nt)	sense seed match (2-7nt)	antisense seed match (2-7nt)
17503937	Mt2	2.88	0.0000027	0.023	0	0	0	0
17503942	Mt1	2.38	0.0003358	0.179	0	0	0	0
17424088	Mir5123	1.76	0.0003772	0.181	NA	NA	NA	NA
17229036	Fmo2	1.51	0.0001888	0.159	1	0	0	0
17337816	Cyp39a1	1.34	0.0007026	0.212	6	1	0	0
17516938	Bud13	1.32	0.0000228	0.067	1	0	0	0
17449553	Ankrd17	1.25	0.0005976	0.205	NA	NA	NA	NA
17274889	Gdap10	1.22	0.0000162	0.061	NA	NA	NA	NA
17516921	Zpr1	1.17	0.0000021	0.022	2	1	0	1
17337314	Ppp1r10	1.11	0.0000263	0.072	0	1	0	0
17371310	B3galt1	1.01	0.0002366	0.166	12	2	1	0
17278321	Serpina3m	1.00	0.0004524	0.191	1	0	0	0
17289275	Snora47	0.96	0.0000162	0.061	NA	NA	NA	NA
17468417	Cml3	0.96	0.0001421	0.155	1	0	0	0
17240819	Cep85l	0.92	0.0000784	0.120	4	0	NA	NA
17361805	Neat1	0.92	0.0007471	0.214	NA	NA	NA	NA
17222527	Tbc1d8	0.91	0.0004057	0.184	8	1	0	0
17454193	Cyp3a13	0.83	0.0002528	0.166	1	0	0	0
17238191	BC089597	0.81	0.0000192	0.066	0	0	0	0
17485702	Leng9	0.80	0.0000002	0.005	0	0	0	0
17243659	Gm6713	0.80	0.0000873	0.129	NA	NA	NA	NA
17353141	Mir187	0.80	0.0009181	0.234	0	0	NA	NA
17511660	Ces1b	0.76	0.0007980	0.223	0	0	0	0
17265164	Bcl6b	0.73	0.0009865	0.244	1	0	0	0
17252183	Eno3	0.73	0.0000675	0.116	0	0	0	0
17341076	4930474M22Rik	0.72	0.0001356	0.155	NA	NA	NA	NA
17455159	Cyp3a44	0.72	0.0001741	0.155	1	0	0	0
17461341	Itpr1	0.71	0.0001374	0.155	15	2	0	0
17534074	Lonrf3	0.71	0.0001298	0.155	3	0	0	0
17307080	Pspc1	0.70	0.0008387	0.226	1	1	0	0
17550450	Snhg1	0.70	0.0007620	0.214	NA	NA	NA	NA
17516915	Apoa5	0.69	0.0000114	0.061	0	0	0	0
17242578	Ppap2c	0.67	0.0000585	0.108	0	1	0	0
17337387	2410017I17Rik	0.64	0.0010336	0.248	3	1	0	0
17462139	Olfr213	0.64	0.0008697	0.228	0	0	NA	NA
17349554	Matr3	0.64	0.0004022	0.184	1	0	0	0
17466025	Klrg2	0.61	0.0003520	0.180	0	1	0	0
17269717	Stat3	0.61	0.0009565	0.240	9	0	0	0
17441999	Fam109a	0.60	0.0006921	0.210	2	0	0	0
17382378	D530008I23	0.60	0.0006609	0.210	NA	NA	NA	NA
17373781	Lmo2	0.59	0.0008486	0.226	3	0	0	0
17476856	E130304I02Rik	0.59	0.0005448	0.200	NA	NA	NA	NA
17350243	Spink6	0.57	0.0004247	0.187	0	0	0	0
17527261	Cib2	0.57	0.0007468	0.214	2	0	0	0
17320928	Slc38a2	0.56	0.0004254	0.187	1	0	0	0
17276070	Arid4a	0.54	0.0009856	0.244	6	3	0	0
17463478	BC064078	0.52	0.0004495	0.191	NA	NA	NA	NA
17507940	Defb9	0.52	0.0001107	0.148	0	0	0	0
17290195	Gm20767	0.51	0.0007226	0.213	0	0	NA	NA
17365960	Gfra1	0.50	0.0002959	0.177	15	2	0	0
17452713	Vps37b	0.50	0.0002416	0.166	4	0	1	0
17510247	Rpl18a	0.50	0.0008333	0.226	2	0	0	0
17282420	Zfyve1	0.48	0.0005802	0.205	8	0	0	0
17271005	Smurf2	0.47	0.0006203	0.207	4	1	0	0
17239493	Heca	0.46	0.0007228	0.213	0	0	0	0
17550522	Peril	0.45	0.0008191	0.224	NA	NA	NA	NA
17264342	Glp2r	0.43	0.0008731	0.228	6	1	1	0
17509723	Psd3	0.42	0.0009459	0.238	16	3	0	0
17425836	Ptbp3	0.39	0.0005515	0.200	5	0	0	0
17221360	Lactb2	-0.41	0.0010049	0.246	4	0	0	0
17272396	Prpsap1	-0.41	0.0005850	0.205	5	2	0	0
17365953	B230217O12Rik	-0.46	0.0002397	0.166	4	1	NA	NA
17503507	Lonp2	-0.48	0.0006026	0.205	5	0	0	0
17408289	Gm4450	-0.48	0.0006686	0.210	2	0	0	0
17352603	Usp14	-0.48	0.0001974	0.160	3	1	0	0
17548711	Gm10690	-0.51	0.0003823	0.181	NA	NA	NA	NA

DOWN-REGULATED

17327465	Ets2	-0.51	0.0002528	0.166	2	0	0	0
17233565	Micu1	-0.53	0.0006656	0.210	12	2	0	0
17401086	Sike1	-0.53	0.0005780	0.205	0	1	0	0
17510885	Elmod2	-0.53	0.0001530	0.155	1	1	0	0
17517741	Scamp2	-0.53	0.0004692	0.192	3	0	0	0
17346956	Myl12b	-0.54	0.0007608	0.214	3	0	0	0
17474052	Kptn	-0.54	0.0007104	0.213	1	1	0	0
17334932	Ergic1	-0.55	0.0000345	0.076	10	2	1	0
17515277	Smarca4	-0.56	0.0003431	0.180	7	1	0	0
17525993	Tbcel	-0.57	0.0002899	0.177	10	1	0	0
17472598	C2cd5	-0.58	0.0010100	0.246	8	0	0	0
17211223	Xkr9	-0.60	0.0005739	0.205	0	0	0	0
17225993	Rnf152	-0.61	0.0009351	0.237	2	1	0	0
17499290	2810030D12Rik	-0.61	0.0001983	0.160	NA	NA	NA	NA
17233708	Lrrc20	-0.61	0.0006712	0.210	7	1	1	1
17423893	Smim8	-0.62	0.0002356	0.166	1	0	0	0
17525955	Sc5d	-0.64	0.0002072	0.160	3	0	0	0
17251454	Ctc1	-0.65	0.0006856	0.210	3	0	1	0
17246284	Suox	-0.67	0.0000743	0.120	0	0	0	0
17507082	Pex11g	-0.67	0.0003277	0.179	1	0	0	0
17241756	C730027H18Rik	-0.68	0.0008199	0.224	NA	NA	NA	NA
17320538	Arsa	-0.69	0.0003191	0.179	0	0	0	0
17323755	Cldn5	-0.69	0.0004816	0.193	0	0	0	0
17231844	Perp	-0.70	0.0007292	0.214	4	0	0	0
17519598	Elovl5	-0.72	0.0003821	0.181	2	2	0	0
17276985	Sipa1l1	-0.72	0.0001209	0.151	20	3	0	0
17548153	Cat	-0.73	0.0007609	0.214	NA	NA	NA	NA
17515539	Eepd1	-0.74	0.0003480	0.180	8	3	0	0
17319106	Gm17753	-0.74	0.0000601	0.108	NA	NA	NA	NA
17250129	2810021J22Rik	-0.75	0.0010434	0.249	1	0	0	0
17517112	Sdhd	-0.76	0.0004649	0.192	NA	NA	NA	NA
17252912	Tlcd2	-0.77	0.0002503	0.166	0	0	0	0
17478301	Nav2	-0.79	0.0004784	0.193	56	5	3	0
17548715	Arcn1	-0.79	0.0006567	0.210	NA	NA	NA	NA
17505148	Cdh1	-0.80	0.0009080	0.233	6	2	1	0
17464573	Tfpi2	-0.80	0.0003851	0.181	0	0	0	0
17475026	Ethe1	-0.83	0.0001764	0.155	1	1	0	0
17441051	Oasl1	-0.85	0.0006309	0.209	2	0	0	0
17249524	Shroom1	-0.85	0.0001161	0.150	1	0	0	0
17224540	Tuba4a	-0.91	0.0001722	0.155	NA	NA	NA	NA
17410400	Cyp2u1	-0.92	0.0000001	0.003	1	0	0	0
17449521	Cox18	-0.93	0.0001760	0.155	NA	NA	NA	NA
17326987	Mrap	-0.93	0.0008467	0.226	0	0	0	0
17252689	Spata22	-0.95	0.0005082	0.197	0	0	0	0
17302074	Tsc22d1	-1.00	0.0000152	0.061	9	4	0	0
17400000	S100a10	-1.01	0.0002084	0.160	2	0	0	0
17357552	Ddb1	-1.07	0.0005403	0.200	NA	NA	NA	NA
17289160	Gm15622	-1.10	0.0005108	0.197	NA	NA	NA	NA
17416154	Gm12718	-1.14	0.0004451	0.191	8	1	NA	NA
17407956	Car14	-1.19	0.0000139	0.061	NA	NA	NA	NA
17397932	Sucnr1	-1.19	0.0000219	0.067	0	0	0	0
17359929	Gbf1	-1.24	0.0002875	0.177	NA	NA	NA	NA
17379169	Sgk2	-1.39	0.0002771	0.176	3	1	0	0
17233064	Atg5	-1.41	0.0000350	0.076	NA	NA	NA	NA
17268884	Nr1d1	-1.43	0.0004303	0.187	4	0	0	0
17482897	Aqp8	-1.49	0.0000583	0.108	1	0	0	0
17364208	Ppp1r3c	-1.71	0.0003344	0.179	1	1	1	0
17233645	Psap	-2.20	0.0005364	0.200	NA	NA	NA	NA
17288876	Arrdc3	-2.20	0.0000009	0.013	1	0	0	0
17391450	Anapc1	-2.67	0.0004097	0.184	NA	NA	NA	NA

Appendix Table S3 - **List of dysregulated genes in shHBV7-treated animals.**
Shown are significantly dysregulated genes in the respective treatment group compared to mock-treated controls. p-values and adjusted p-values (false discovery rate) were controlled by Benjamini-Hochberg and defined as described in Methods. The four columns on the right show the number of shHBV7 sense- or antisense-strand seed matches in the 3'UTR or the whole transcript of the respective gene. NA, non-applicable (because the RNA is non-coding or the sequence could not be retrieved).

Resource / database	Biological process	Genes	%	p-value	Involved genes	Fold enrichment
GOTERM_BP_5	GO:0007263~nitric oxide mediated signal transduction	2	5.71	0.0039	NM_013602, NM_008630	490.62
GOTERM_BP_FAT	GO:0007263~nitric oxide mediated signal transduction	2	5.71	0.0040	NM_013602, NM_008630	481.70
GOTERM_BP_FAT	GO:0010273~detoxification of copper ion	2	5.71	0.0060	NM_013602, NM_008630	321.14
GOTERM_BP_5	GO:0006694~steroid biosynthetic process	3	8.57	0.0081	NM_133748, NM_018887, NM_178082, NM_145942	21.03
GOTERM_BP_FAT	GO:0008203~cholesterol metabolic process	3	8.57	0.0083	NM_133748, NM_018887, NM_178082, NM_145942	20.94
GOTERM_BP_FAT	GO:0006694~steroid biosynthetic process	3	8.57	0.0085	NM_133748, NM_018887, NM_178082, NM_145942	20.64
GOTERM_BP_5	GO:0030217~T cell differentiation	3	8.57	0.0088	NM_007913, NM_011817, NM_011281	20.16
GOTERM_BP_FAT	GO:0030217~T cell differentiation	3	8.57	0.0093	NM_007913, NM_011817, NM_011281	19.80
GOTERM_BP_5	GO:0016125~sterol metabolic process	3	8.57	0.0095	NM_133748, NM_018887, NM_178082, NM_145942	19.37
GOTERM_BP_FAT	GO:0016125~sterol metabolic process	3	8.57	0.0100	NM_133748, NM_018887, NM_178082, NM_145942	19.01
GOTERM_BP_5	GO:0006916~anti-apoptosis	3	8.57	0.0126	NM_010478, NM_011415, NM_010849	16.73
GOTERM_BP_FAT	GO:0006916~anti-apoptosis	3	8.57	0.0132	NM_010478, NM_011415, NM_010849	16.42
GOTERM_BP_5	GO:0046688~response to copper ion	2	5.71	0.0136	NM_013602, NM_008630	140.18
GOTERM_BP_FAT	GO:0046688~response to copper ion	2	5.71	0.0139	NM_013602, NM_008630	137.63
GOTERM_BP_FAT	GO:0006882~cellular zinc ion homeostasis	2	5.71	0.0139	NM_013602, NM_008630	137.63
GOTERM_BP_FAT	GO:0055069~zinc ion homeostasis	2	5.71	0.0159	NM_013602, NM_008630	120.43
GOTERM_BP_FAT	GO:0008610~lipid biosynthetic process	4	11.43	0.0178	NM_133748, NM_018887, NM_178082, NM_145942, NM_007703	6.88
GOTERM_BP_5	GO:0030098~lymphocyte differentiation	3	8.57	0.0193	NM_007913, NM_011817, NM_011281	13.38
GOTERM_BP_FAT	GO:0030098~lymphocyte differentiation	3	8.57	0.0202	NM_007913, NM_011817, NM_011281	13.14
GOTERM_BP_5	GO:0042110~T cell activation	3	8.57	0.0203	NM_007913, NM_011817, NM_011281	13.03
GOTERM_BP_FAT	GO:0042110~T cell activation	3	8.57	0.0212	NM_007913, NM_011817, NM_011281	12.79
GOTERM_BP_5	GO:0045884~regulation of survival gene product expression	2	5.71	0.0270	NM_011415, NM_010849	70.09
GOTERM_BP_5	GO:0042474~middle ear morphogenesis	2	5.71	0.0270	NM_133748, NM_178082, NM_010849	70.09
GOTERM_BP_FAT	GO:0042474~middle ear morphogenesis	2	5.71	0.0276	NM_133748, NM_178082, NM_010849	68.81
GOTERM_BP_FAT	GO:0045884~regulation of survival gene product expression	2	5.71	0.0276	NM_011415, NM_010849	68.81
GOTERM_BP_FAT	GO:0044092~negative regulation of molecular function	3	8.57	0.0279	NM_010478, NM_010849, NM_011817	11.03
GOTERM_BP_FAT	GO:0030005~cellular di-, tri-valent inorganic cation homeostasis	3	8.57	0.0287	NM_013602, NM_008630, NM_001111288	10.87
GOTERM_BP_5	GO:0002521~leukocyte differentiation	3	8.57	0.0294	NM_007913, NM_011817, NM_011281	10.67
GOTERM_BP_FAT	GO:0002521~leukocyte differentiation	3	8.57	0.0308	NM_007913, NM_011817, NM_011281	10.47
GOTERM_BP_FAT	GO:0055066~di-, tri-valent inorganic cation homeostasis	3	8.57	0.0337	NM_013602, NM_008630, NM_001111288	9.97
GOTERM_BP_5	GO:0030003~cellular cation homeostasis	3	8.57	0.0342	NM_013602, NM_008630, NM_001111288	9.81
GOTERM_BP_FAT	GO:0030003~cellular cation homeostasis	3	8.57	0.0358	NM_013602, NM_008630, NM_001111288	9.63
GOTERM_BP_FAT	GO:0046632~alpha-beta T cell differentiation	2	5.71	0.0373	NM_011817, NM_011281	50.71
GOTERM_BP_5	GO:0008202~steroid metabolic process	3	8.57	0.0376	NM_133748, NM_018887, NM_178082, NM_145942	9.32
GOTERM_BP_FAT	GO:0008202~steroid metabolic process	3	8.57	0.0394	NM_133748, NM_018887, NM_178082, NM_145942	9.15
GOTERM_BP_5	GO:0046631~alpha-beta T cell activation	2	5.71	0.0440	NM_011817, NM_011281	42.66
GOTERM_BP_5	GO:0006695~cholesterol biosynthetic process	2	5.71	0.0440	NM_133748, NM_178082, NM_145942	42.66
GOTERM_BP_FAT	GO:0006695~cholesterol biosynthetic process	2	5.71	0.0450	NM_133748, NM_178082, NM_145942	41.89
GOTERM_BP_FAT	GO:0046631~alpha-beta T cell activation	2	5.71	0.0450	NM_011817, NM_011281	41.89
GOTERM_BP_FAT	GO:0055080~cation homeostasis	3	8.57	0.0508	NM_013602, NM_008630, NM_001111288	7.94
GOTERM_BP_FAT	GO:0046649~lymphocyte activation	3	8.57	0.0513	NM_007913, NM_011817, NM_011281	7.90
GOTERM_BP_FAT	GO:0009636~response to toxin	2	5.71	0.0564	NM_013602, NM_008630	33.22
GOTERM_BP_5	GO:0016126~sterol biosynthetic process	2	5.71	0.0570	NM_133748, NM_178082, NM_145942	32.71
GOTERM_BP_FAT	GO:0016126~sterol biosynthetic process	2	5.71	0.0583	NM_133748, NM_178082, NM_145942	32.11
PANTHER_PATHWAY	P05914:Nicotine degradation	2	5.71	0.0612	NM_018881, NM_009997	28.95
GOTERM_BP_FAT	GO:0045321~leukocyte activation	3	8.57	0.0649	NM_007913, NM_011817, NM_011281	6.91
GOTERM_BP_FAT	GO:0051726~regulation of cell cycle	3	8.57	0.0671	NM_010831, NM_010849, NM_011817	6.78
KEGG_PATHWAY	mmu05020:Prion diseases	2	5.71	0.0765	NM_010478, NM_007913	23.45
GOTERM_BP_5	GO:0043066~negative regulation of apoptosis	3	8.57	0.0774	NM_010478, NM_011415, NM_010849	6.21
GOTERM_BP_FAT	GO:0001775~cell activation	3	8.57	0.0802	NM_007913, NM_011817, NM_011281	6.12
GOTERM_BP_5	GO:0043069~negative regulation of programmed cell death	3	8.57	0.0802	NM_010478, NM_011415, NM_010849	6.08
GOTERM_BP_FAT	GO:0043066~negative regulation of apoptosis	3	8.57	0.0807	NM_010478, NM_011415, NM_010849	6.10
GOTERM_BP_5	GO:0060548~negative regulation of cell death	3	8.57	0.0808	NM_010478, NM_011415, NM_010849	6.06
GOTERM_BP_5	GO:0030097~hemopoiesis	3	8.57	0.0814	NM_007913, NM_011817, NM_011281	6.03
GOTERM_BP_FAT	GO:0043069~negative regulation of programmed cell death	3	8.57	0.0837	NM_010478, NM_011415, NM_010849	5.97
GOTERM_BP_FAT	GO:0009628~response to abiotic stimulus	3	8.57	0.0843	NM_010478, NM_011415, NM_010849	5.95
GOTERM_BP_FAT	GO:0060548~negative regulation of cell death	3	8.57	0.0843	NM_010478, NM_011415, NM_010849	5.95
GOTERM_BP_FAT	GO:0030097~hemopoiesis	3	8.57	0.0849	NM_007913, NM_011817, NM_011281	5.92
KEGG_PATHWAY	mmu05219:Bladder cancer	2	5.71	0.0936	NM_010849, NM_138302	18.98
GOTERM_BP_FAT	GO:0006873~cellular ion homeostasis	3	8.57	0.0940	NM_013602, NM_008630, NM_001111288	5.58
GOTERM_BP_5	GO:0055082~cellular chemical homeostasis	3	8.57	0.0943	NM_013602, NM_008630, NM_001111288	5.53
GOTERM_BP_5	GO:0052547~regulation of peptidase activity	2	5.71	0.0951	NM_010478, NM_010849	19.24
GOTERM_BP_FAT	GO:0052548~regulation of endopeptidase activity	2	5.71	0.0954	NM_010478, NM_010849	19.27
GOTERM_BP_FAT	GO:0043281~regulation of caspase activity	2	5.71	0.0954	NM_010478, NM_010849	19.27
GOTERM_BP_FAT	GO:0052547~regulation of peptidase activity	2	5.71	0.0972	NM_010478, NM_010849	18.89
GOTERM_BP_5	GO:0048534~hemopoietic or lymphoid organ development	3	8.57	0.0979	NM_007913, NM_011817, NM_011281	5.41
GOTERM_BP_FAT	GO:0055082~cellular chemical homeostasis	3	8.57	0.0983	NM_013602, NM_008630, NM_001111288	5.43
GOTERM_BP_FAT	GO:0010038~response to metal ion	2	5.71	0.0990	NM_013602, NM_008630	18.53

Appendix Table S4 - List of dysregulated pathways in shHBV7-treated animals.
Pathway enrichment of genes significantly dysregulated in shHBV7-treated animals compared to the empty control vector was obtained by querying pathway annotations using the R-package RDAVIDWebService as described in Methods.

Name	Sequence (5´ to 3´)	Used for
eGFP fwd	GAGCGCACCATCTTCTTCAAG	Vector titration
eGFP rev	TGTCGCCCTCGAACTTCAC	
eGFP probe	FAM-ACGACGGCAACTACA-BHQ1	
TTR fwd	TGTTCCGATACTCTAATCTCCC	Vector titration
TTR rev	TATACCCCCTCCTTCCAACC	
TTR probe	FAM-TTTGGAGTCAGCTTGGCAGGGATCA-BHQ1	
SV40 p(A) SalI fwd	TACT GCTAGT CAGACATGATAAGATACATTGATG	Transfer of SV40/Poly A into pBS-H1-hAAT19
SV40 p(A) BamHI rev	TACTT GGATCC CCACATTTGTAGAGGTTTTACTTGC	
shHBV4 fwd	CACC GGCTCAGTTTACTAGTGCCATT TCAAGAG AATGGCACTAGTAAACTGAGCC	Insertion of shRNA in expression vector
shHBV4 rev	AAAA GGCTCAGTTTACTAGTGCCATT CTCTTGA AATGGCACTAGTAAACTGAGCC	
shHBV5 fwd	CACC GCCAAAATTCGCAGTCCCCAAC TCAAGAG GTTGGGGACTGCGAATTTTGGC	Insertion of shRNA in expression vector
shHBV5 rev	AAAA GCCAAAATTCGCAGTCCCCAAC CTCTTGA GTTGGGGACTGCGAATTTTGGC	
shHBV6 fwd	CACC GGACTCTTGGACTCTCAGCAAT TCAAGAG ATTGCTGAGAGTCCAAGAGTCC	Insertion of shRNA in expression vector
shHBV6 rev	AAAA GGACTCTTGGACTCTCAGCAAT CTCTTGA ATTGCTGAGAGTCCAAGAGTCC	
shHBV7 fwd	CACC GCGCTGAATCCTGCGGACGACC TCAAGAG GGTCGTCCGCAGGATTCAGCGC	Insertion of shRNA in expression vector
shHBV7 rev	AAAA GCGCTGAATCCTGCGGACGACC CTCTTGA GGTCGTCCGCAGGATTCAGCGC	
TTR SpeI fwd	AATT ACTAGT GGATCTGTCAATTCACGCGAG	Insertion of TTR into Ago2 co-expression vector
TTR SacI rev	CATG GAGCTC CAGCTGGGCTTCTCCTGGTG	
a = hcr XhoI fwd	GAGTC CTCGAG TGGAGGTGAAGTTAACACCTTCGTG	1st and 3rd PCR pri-miR122/HBV7 overlap extension PCR
b = miRHBV7 rev	GCTT ATAGTTTAGACAAAGCGCTGAATCCTGCGGACGACAGCTCTGCTAAGG	1st PCR pri-miR122/HBV7 overlap extension PCR
c = miRHBV7 fwd	GCTT TGTCTAAACTATAAGCGCTGAATaCTGCGGAatATAGCTACTGCTAGGC	2nd PCR pri-miR122/HBV7 overlap extension PCR
d = hcr NheI rev	CTCG GCTAGC AAGCAAACGATGCCAAGACATTTATCG	2nd and 3rd PCR pri-miR122/HBV7 overlap extension PCR
TuDHBV7 fwd	AATACGTCTCGTCCCA GGTCGTCCGCAGGATTCAGCGC CAAGTATTCTGGTCACAGAATA	Insertion of TuDHBV7 in expression vector
TuDHBV7 rev	AATACGTCTCGTCGGT GCGCTGAATCCTGCGGACGACC GTTGTATTCTGTGACCAGAATA	
TuDα1AT fwd	AATACGTCTCGTCCCA TTAAACATGCCTAAACGCTTCCAAGTATTCTGGTCACAGAATA	Insertion of TuDα1AT in expression vector
TuDα1AT rev	AATACGTCTCGTCGGT GAAGCGTTTAGGCATGTTTAACGTTGTATTCTGTGACCAGAATA	
shHBV7 anti-sense Fw XhoI	TCGA AAGCGCTGAATCCTGCGGACGA	shHBV7 antisense luciferase reporter
shHBV7 anti-sense Rev NotI	GGCC TCGTCCGCAGGATTCAGCGCTT	
shHBV7 sense Fw XhoI	TCGA GGTCGTCCGCAGGATTCAGCGC	shHBV7 sense luciferase reporter
shHBV7 sense Rev NotI	GGCC GCGCTGAATCCTGCGGACGACC	
HBV 3.5kb RNA Fw	GAGTGTGGATTCGCACTCC	HBV 3.5kb transcript
HBV 3.5kb RNA Rev	GAGGCGAGGGAGTTCTTCT	
PCR conditions	Denaturation: 95°C 5 min; Amplification: 95°C 03s, 60°C 30s (40 cycles)	
HBV total RNA Fw	TCACCAGCACCATGCAAC	HBV total RNA
HBV total RNA Rev	AAGCCACCCAAGGCACAG	
PCR conditions	Denaturation: 95°C 5 min; Amplification: 95°C 03s, 60°C 30s (40 cycles)	
B2M 1 Fw	GCTATCCAGCGTACTCCAAAGAT	Beta-2-microglobulin (housekeeping gene for cell culture experiments)
B2M 1 Rev	GCTTACATGTCTCGATCCCACT	
PCR conditions	Denaturation: 95°C 5 min; Amplification: 95°C 15s, 60C° 10s, 72°C 25s (45 cycles)	
HBV rcDNA selective Fw	TACTAGGAGGCTGTAGGCATA	HBV rcDNA selective primer (cell culture experiments)
HBV rcDNA selective Rev	GGAGACTCTAAGGCTTCCC	
PCR conditions	Denaturation: 95°C 5 min; Amplification: 95°C 25s, 60C° 15s, 72°C 30s (45 cycles)	
HBV 1844	GTTGCCCGTTTGTCCTCTAATTC	HBV DNA (mouse experiment)
HBV 1745	GGAGGGATACATAGAGGTTCCTTGA	
PCR conditions	Denaturation: 95°C 5 min; Amplification: 95°C 25s, 60°C 15s, 72°C 30s (45 cycles)	
mCyclophilin Fw	ATGGTCAACCCCACCGTGT	Housekeeping gene for mouse experiment
mCyclophilin Rev	TTCTGCTGTCTTTGGAACTTTGTC	
PCR conditions	Denaturation: 95°C 10 min; Amplification: 95°C 15s, 60°C 15s (45 cycles)	
mHPRT Fw	CTGATTATGGACAGGACTGAAAGA	Murine Hypoxanthine-guanine phosphoribosyltransferase (housekeeping gene for mouse experiment)
mHPRT Rev	CCGTTGACTGATCATTACAGTAGC	
PCR conditions	Denaturation: 95°C 10 min; Amplification: 95°C 15s, 60°C 15s (45 cycles)	

Appendix Table S5 - **List of oligonucleotides including PCR conditions used in this study.**
BHQ1, Black Hole Quencher 1; FAM, Carboxyfluorescein; Fwd/Fw, forward; rev/Rev, reverse; B2M, Beta-2-microglobulin; mHPRT, murine Hypoxanthine-guanine phosphoribosyltransferase.
