# Supplementary material for: Blocking sense‐strand activity improves potency, safety and specificity of anti‐hepatitis B virus short hairpin RNA
Source: EMBO Mol Med. 2016 Jul 29;8(9):1082–98. doi: 10.15252/emmm.201506172 (PMC5009812; doi:10.15252/emmm.201506172)
Supplement: Supplementary file 2 — Expanded View Figures PDF [file EMMM-8-1082-s002.pdf]

Expanded View Figures

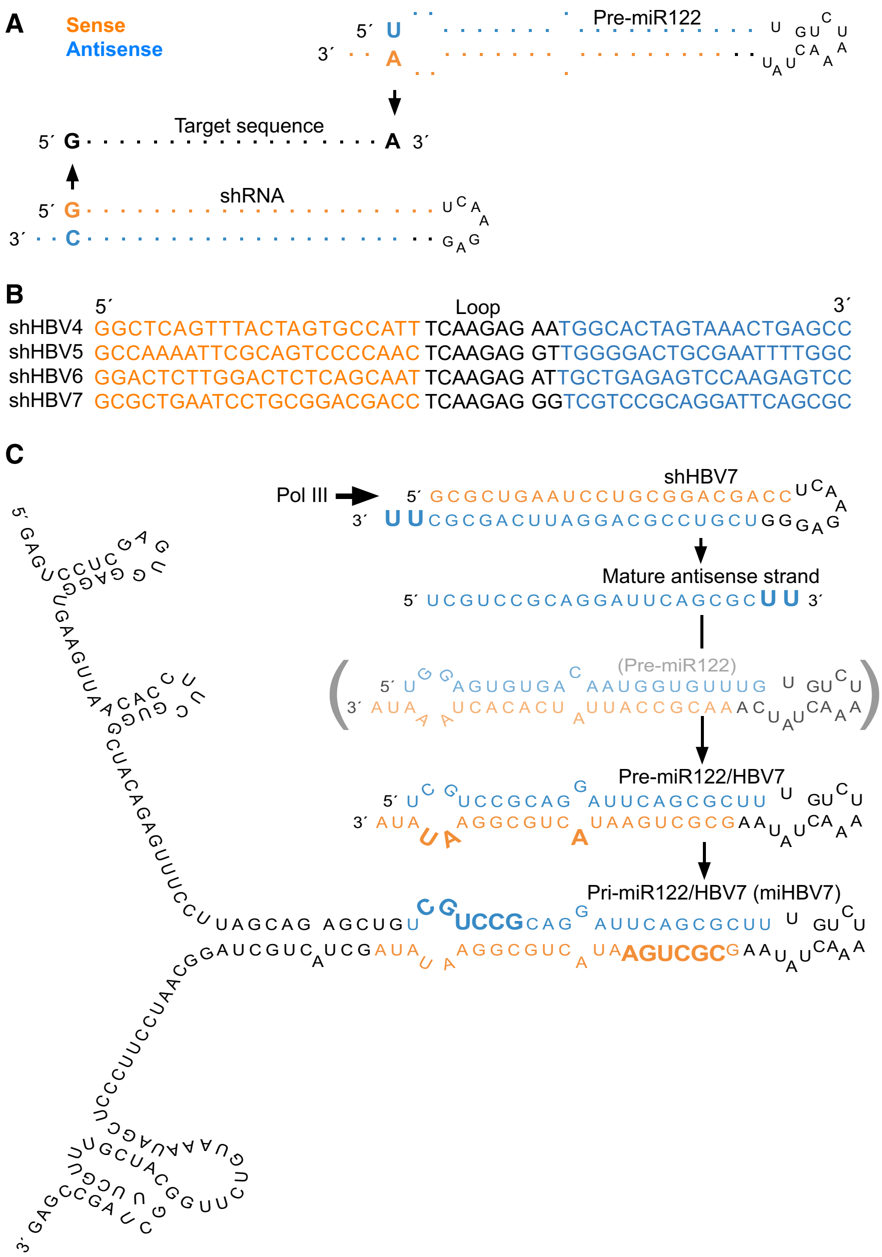

Figure EV1. Design of anti-HBV shRNAs and pri-miRNA mimic.

A HBV target sequence (black) and corresponding pre-miR-122-embedded (top) or conventional (bottom) shRNAs. The selected HBV target sequences had to allow expression as classical shRNA or from a miRNA scaffold, and TuD-mediated sense-strand neutralisation.

B Four selected shRNAs targeting HBV sequences that fulfil all requirements from panel (A).

C Stepwise derivation of a pri-miR-122-embedded anti-HBV hairpin from shHBV7 (lead candidate from panel B). Note that shHBV7 and pri-miR-122/HBV7 are designed to encode the identical antisense strand (blue) against HBV, and that the seed region of the sense strand of pri-miR-122/HBV7 is identical to the seed region of the shHBV7 sense strand (bold letters).

 © 2016 The Authors

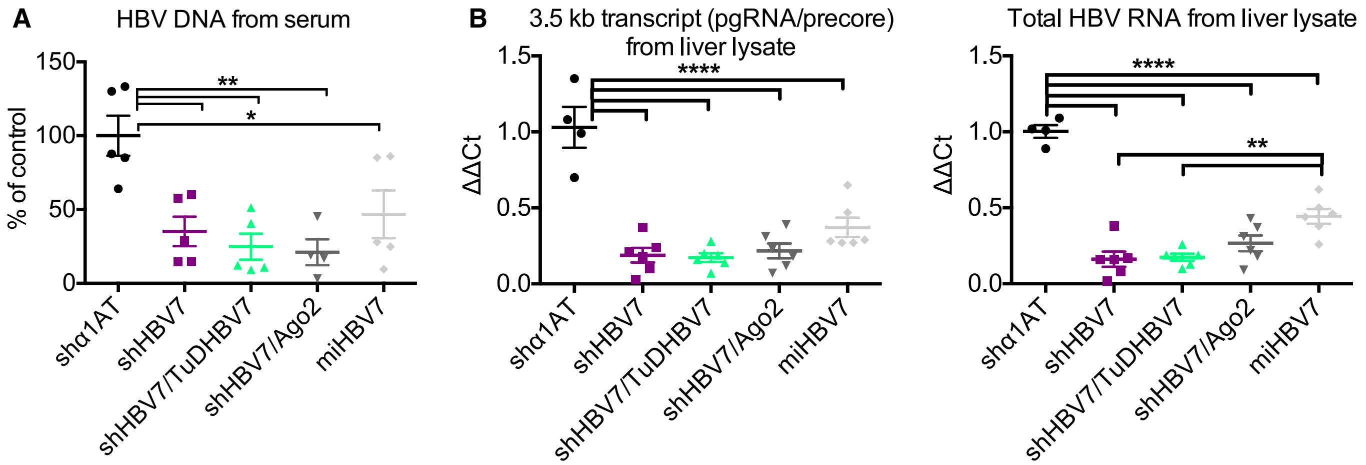

Figure EV2. Effect of different RNAi strategies on HBV DNA in serum and HBV transcripts in livers of HBV-transgenic mice.

A HBV-transgenic mice were injected i.v. with 1×10^{11} particles of the shown AAV vectors. HBV DNA was measured at day 84 after treatment.

B Expression of HBV transcripts in livers of HBV-transgenic mice was analysed at day 15 after treatment.

Data information: Mean values and SEM are shown, and significance was calculated using one-way ANOVA with Tukey's multiple comparison correction. $^*P < 0.05$; $^{**}P < 0.01$; $^{****}P < 0.0001$. See Appendix Table S1 for exact *n*- and *P*-values.

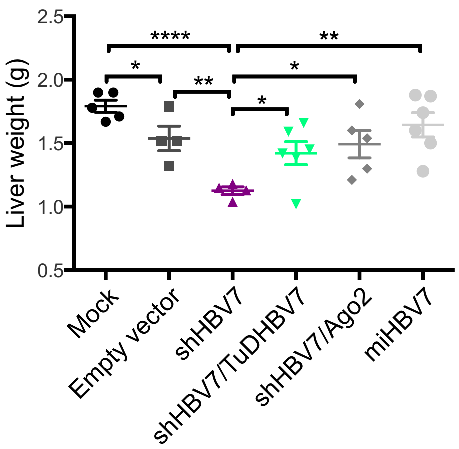

Figure EV3. Liver weight from short-term *in vivo* study.

HBV-transgenic mice (same mice as in Fig 5) were injected i.v. with 1×10^{11} particles of the shown AAV vectors, and their livers were harvested and weighed 15 days later. Mean values and SEM are shown, and significance was calculated using Student *t*-test. $^*P < 0.05$; $^{**}P < 0.01$; $^{****}P < 0.0001$. See Appendix Table S1 for exact *n*- and *P*-values.

© 2016 The Authors

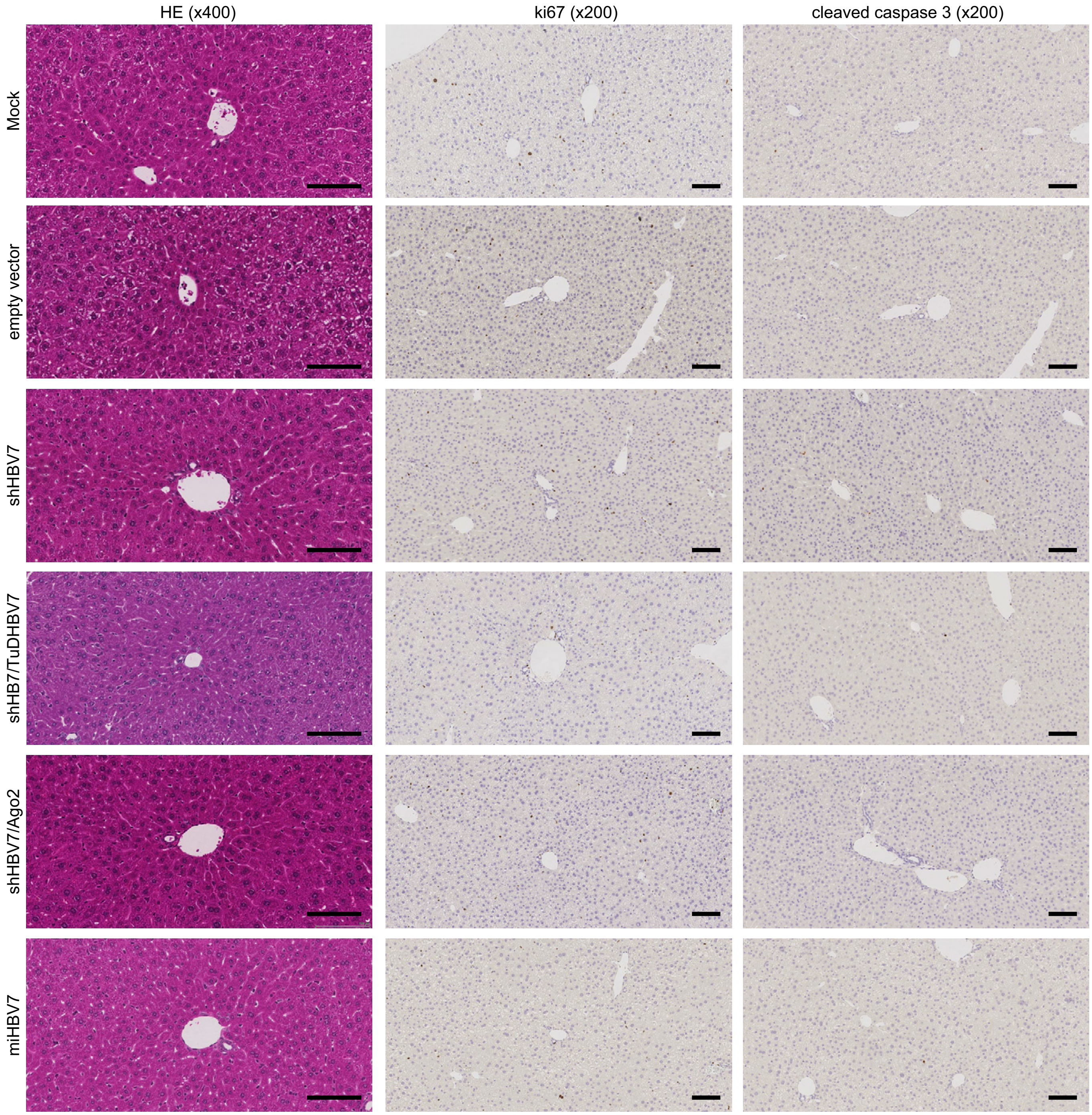

Figure EV4. Pathohistological stainings of livers of HBV-transgenic mice.
Livers were harvested 15 days after intravenous injection of 1×10^{11} particles of the vectors shown on the left. HE, haematoxylin/eosin; ki67, cellular proliferation marker. ×400/×200, 400- or 200-fold magnification, respectively. Scale bar at the bottom right always represents 100 μm.

 © 2016 The Authors

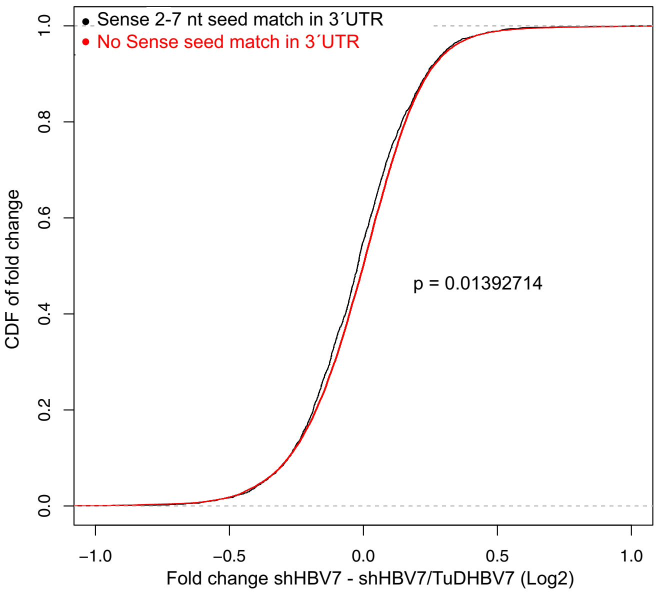

Figure EV5. Specific rescue of shHBV7 sense targets.
Cumulative distribution of fold changes (CDF analysis) between shHBV7- and shHBV7/TuDHBV7-treated animals was calculated for genes with 3′UTR 2- to 7-nt sense-strand seed match (black line) and for genes without this match (red line). Statistical analysis was performed using a two-sample, two-sided Welch's *t*-test.

© 2016 The Authors
